# Natural disaster and medication preparedness among elderly: a scoping review

**DOI:** 10.12688/f1000research.157483.2

**Published:** 2025-06-02

**Authors:** Rika Yuliwulandari, Rifda El Mahroos, Zulfan Febriawan, Adi Wibowo, Debrina Kusuma Devi, Eko Teguh Paripurno, Awang Hendrianto Pratomo, Tedy Agung Cahyadi, Yohana Noradika Maharani, Johan Danu Prasetya, Reza Pahlevi Ramadhani Arfindra Setiawan, Hafiz T.A Khan

**Affiliations:** 1 Department of Pharmacology, Faculty of Medicine, Universitas Pembangunan Nasional “Veteran” Jawa Timur, Surabaya, East Java, 60294, Indonesia; 2 Department of Physiology, Faculty of Medicine, Universitas Pembangunan Nasional “Veteran” Jawa, Surabaya, East Java, 60294, Indonesia; 3 Department of Informatics, Faculty of Science and Mathematics, Universitas Diponegoro, Semarang, Central Java, 50275, Indonesia; 4 Department of Internal Medicine, Universitas Pembangunan Nasional “Veteran” Jawa Timur, Surabaya, East Java, 55283, Indonesia; 5 Master Program of Disaster Management, Faculty of Mineral Technology, Universitas Pembangunan Nasional Veteran Yogyakarta, Yogyakarta, 55283, Indonesia; 6 Faculty of Medicine, Universitas Pembangunan Nasional Veteran Jawa Timur, Surabaya, East Java, 60294, Indonesia; 7 Public Health, College of Nursing, Midwifery, and Healthcare, University of West London, London, England, W5 5RF, UK; 8 Oxford Institute of Population Ageing, University of Oxford, Oxford, England, UK

**Keywords:** Elderly, Health, Medication, Natural Disaster, Preparedness

## Abstract

**Introduction:**

The increasing number of older people and their vulnerability to disaster and medication preparedness as the primary elements of disaster mitigation are necessary to reduce the impact of a disaster. Nevertheless, research on natural disasters and medication preparedness in the elderly population is still lacking. This review aimed to explore natural disaster and medication preparedness among elderly.

**Methods:**

This review was guided by the Arksey & O’Malley methodological framework and reporting accordance to PRISMA-ScR. A scoping review was performed using the following four databases: Scopus, PubMed, Sage, and Google Scholar. Screening was conducted using the following criteria: articles written in English, open access, and published between 2020 and 2024. Articles must discuss natural disasters and medication preparedness for elderly. In the data search, we input several keywords that include “elderly,” “natural disaster,” “preparedness,” and “medication.” Snowballing was then conducted to find articles on preparedness interventions. Data extraction and analysis were then performed.

**Results:**

There were 20 articles used in this review and the results highlight that elderly face unique challenges in disaster preparedness including, mobility limitation, restricted access to medication, communication barriers, limited social and social support. Tailored interventions such as disaster education and elderly-focused technology are crucial to improve preparedness and ensure their safety during emergencies.

**Conclusion:**

The findings from this literature review are the majority of studies showing that most elderly people are not well prepared in facing disasters; however, through various programs that have been implemented by either the government or community, the elderly show more preparation when they encounter any natural disaster.

## Introduction

Aging is a phase of the life cycle encountered by everyone that result in a group of population classified as elderly. ^1^ The global population of elderly people, defined as those 60 years of age or greater, is large and increasing. There were an estimated 1 billion elderly people alive in 2024, a group that is projected to grow to 1.4 billion in 2030 and 2.1 billion by 2050. ^2^ Elderly people experience many physiological changes in various organs that make them become vulnerable to health problems and may decreased quality of life (QoL). ^3^ These circumstances can affect their safety and capacity to understand complex medical information. ^4^

The decline in body function and health status of the elderly often becomes an obstacle for them in dealing with emergencies, natural disasters and disease outbreaks that makes them classified as the most vulnerable population group. ^5^ That is because they have reduced ability to prepare, respond and deal with disasters, therefore, they may be more susceptible to injury, death or health problems during and post-disaster. ^6^ The key component of effective disaster mitigation is preparedness. Disaster and medication preparedness is a structured process that includes administrative, organizational, and operational skills to enact strategies and policies, increase management capacity to mitigate the adverse effects of hazards, and decrease the chance of emergencies or disaster effects. Disaster and medication preparedness require collaboration between various stakeholders involving the government, society, and individuals. ^7^

Existing disaster preparedness strategies often focus on the general population and may fail to address the specific needs of older adults. ^8^ Therefore, this article offers a comprehensive review of existing literatures encompassing “elderly natural disaster and medication preparedness.” This review aims to explore natural disaster and medication preparedness among the elderly, including what the elderly need to prepare in facing disaster, as well as the factors that inhibit them from fulfilling the preparedness and certain interventions to support them to be more prepared. Based on available literature, this is the first literature that examines disaster preparedness among the elderly. Hopefully, it can provide important information for policymakers and for any further research.

## Methods

### Database and search approach

This scoping review was guided by the Arksey & O’Malley methodological framework (2005) and reporting accordance with the Preferred Reporting Items for Systematic Reviews and Meta-Analyses Extension for Scoping Reviews (PRISMA-ScR). There are five stages to review the studies include : identifying the question of the studies, identifying some relevant studies, the study selection, charting the data, and the collating, summarizing and reporting the results. Relevant studies were identified using four scientific databases: PubMed (National Center for Biotechnology Information, National Institutes of Health; Bethesda, Maryland, USA); SCOPUS (Elsevier, Amsterdam, Netherlands), Sage Journals (Thousand Oaks, California, USA), and Google Scholar. The search were restricted to : a) studies published from 2020 to April 2024 to identify the updated studies; b) writing in English, and c) primary study (original research and full-text only). The search and review process were conducted by two authors. The keyword searches were as follow:

(“elderly” OR “older people” OR “older adult” OR “old adult” OR “old people” OR “older population”)

AND

(“natural disaster” OR “disaster” OR “emergency” OR “catastrophe” OR “flood” OR “earthquake” OR “hurricane” OR “tsunami”)

AND

(“medication” OR “drug” OR “medicine” OR “prescription”)

AND

(“preparedness” OR “emergency preparedness” OR disaster preparedness” OR “readiness” OR “planning”)

### Eligibility criteria and selection

The screening was conducted in three stages. Initially, two authors independently evaluated the articles based on their titles. The inclusion and exclusion criteria were set based on article type, publication year, access type, language, tier, subject, and age of the participants, as shown in Table 1 (extended data). Papers that did not meet the inclusion criteria were excluded. In the second stage, the abstracts of the selected papers were reviewed. The abstract must contain clear objectives, methods, and results, and also discuss disaster and medication preparedness in the elderly. In the third stage, the full texts of the selected papers were evaluated. The full texts must reveal methodology, reports of the needs and obstacles of elderly people in disaster preparedness, as well as strategies to enhance disaster preparedness in the elderly that include intervention, education, and technology management. The process used to identify the relevant articles for review is shown in Figure 2 (extended data).

### Snowballing

Snowballing involves searching for references and/or citations from articles to identify additional relevant material. The inclusion criteria for this step were similar to those used in the previous step.

### Data extraction and analysis

A data extraction form was used to collect information on each study included in the analysis so that it could be systematically reviewed. The information in the form included the journal author, year of publication, country of origin, sample/sampling technique, research objectives, research design, instrumentation, and major findings. After extraction and structure, the results are discussed.

## Results

### Search results

A total of 920 titles were found across databases (PubMed, Scopus, Sage, and Google Scholar). The titles were then screened based on the inclusion criteria. Duplicated papers were removed, resulting in 163 articles (Figure 1) (extended data). Subsequently, all abstracts were reviewed to determine whether they contained the keywords natural disaster, medication, preparedness, and the elderly. This resulted in 45 articles for further full-text review. Only 14 articles fulfilled the inclusion criteria. Subsequently, snowballing was conducted to identify other relevant materials that contain interventions for disaster preparedness, resulting in six new articles. Thus, the total number of papers included in this study was 20. The final articles eligible for data extraction are listed in Table 2 (extended data).

### Study characteristics

The articles were found mostly conducted in the USA (n=5). ^9– 13^ While other articles were found conducted in several countries such as Indonesia, ^14– 17^ Japan, ^18, 19^ Thailand, ^20, 21^ Puerto Rico, ^22^ Nepal, ^23^ Korea, ^24^ Hong Kong, ^25^ Phillipines, ^26^ and Iran ^27^ (Table 3) (extended data).

### Analysis results

A total of 20 relevant articles were included in this literature review after applying inclusion criteria focused on studies addressing disaster preparedness needs among elderly populations. The analysis of these articles revealed six major categories of disaster preparedness needs: mobility assistance, access to medication and medical supplies, communication support, social support, appropriate shelter and facilities, and tailored education and training programs. Mobility assistance was identified in 1 out of the 20 studies. It reflects the widespread concern over physical limitations among elderly individuals that impede timely evacuation during emergencies. These studies emphasized the necessity of accessible evacuation routes and community-based transport services tailored to elderly users. Access to medication and medical supplies was discussed in 3 studies, underscoring the dependency of older adults on chronic disease medications, which may be disrupted during disasters. Recommendations from the literature include establishing mobile medical units and pre-disaster medication stockpiling. Communication support emerged in 1 studies, particularly noting the barriers elderly individuals face due to hearing or visual impairments. Suggested solutions included visual-based alert systems and text-based notifications that cater to the sensory limitations common in this age group. Social support was addressed in 3 articles, highlighting the importance of family networks, community volunteers, and social programs in ensuring that elderly people receive emotional, physical, and logistical support before, during, and after a disaster. Shelter and special facilities for the elderly were featured in 2 studies, recommending the development of shelters equipped with medical services, trained personnel, and physical infrastructure that accommodate mobility and medical needs. Finally, education and training programs were discussed in 1 articles, emphasizing that elderly individuals often lack the necessary knowledge or confidence to take independent disaster preparedness measures. Several studies recommended community-based educational interventions to build awareness and self-efficacy among the elderly.

## Discussion

Natural disasters such as earthquakes, floods, typhoons, tornadoes, tsunamis, landslides, forest fires, volcanic eruptions, and extreme temperatures cause great damage to the environment. Natural disasters are emergencies that require immediate intervention because of their large impact on human health and safety. ^28^ Elderly people face several different challenges from other age groups when facing these emergencies. Elderly people may encounter challenges such as mobility issues or chronic health conditions during emergencies. Additionally, they may lack nearby family or friends who can offer support during such emergency situations. ^29^ Elderly people have less disaster preparedness and greater vulnerability to disasters. ^8, 9, 18^ About six out of ten elderly people do not consider themselves prepared to face disasters. ^12^ The aims of this review were to explore all reported research and publications related to natural disasters and medication preparedness among the elderly. Among the 20 papers that met all selection criteria, all showed that none of the countries had good preparedness for the elderly when facing a disaster. A USA study by Bell et al. (2021) ^9^ showed that most elderly people are more confident when facing disasters as long as they perform preparedness actions such as a 7-days supply of food and water, a 7-days supply of essential medications/health supplies, a stocked emergency kit, and a conversation about evacuation from home. According to Adepoju et al. (2023), ^12^ six to ten older people in the USA do not perceive themselves as disaster preparedness. In addition, they also suggested the role of home-based providers in improving preparedness and the response of the elderly to disasters. A study in Thailand by Boonyaratkalin (2020) ^20^ showed that 50% of older people exhibit suitable behavior through follow-up news on floods and evacuation planning. Based on a study by Hattori (2020), ^18^ older people are ready to evacuate, especially on confirmed evacuation sites and routes and emergency contact plans; community preparedness, especially on talking with family members about how to evacuate; family preparedness, especially related to fire extinguishers; preparation of emergency goods, especially those related to water, non-perishable foods, and flashlights. However, they are unwell prepared for talking with neighbors on how to evacuate, measures to prevent large electrical applications from moving or falling, footwear for evacuation in the bedroom, portable radio, medical prescription records, medications, and written memos with emergency contact information. In Indonesia, so far, there is not enough data on disaster preparedness in the elderly published in international journals.

Several studies have shown that the majority of the elderly population is not well-prepared for disasters. Therefore, the government, community, and organizations related to disasters have responsibilities to educate elderly people to prepare their needs for disasters, including the following:

### 1. Medication preparedness

The elderly population is vulnerable when a disaster occurs. Many of them have chronic health problems and are highly dependent on drugs and medical equipment. Elderly people with chronic conditions require treatment to survive. In the event of a disaster, it is especially important for elderly people with chronic conditions or other health needs to carry their medicines and medical equipment to reduce health risks and facilitate continuity of their healthcare. The most challenging aspect of the elderly during a disaster is ensuring that they bring all the necessary medications, including medical records, emergency medications, and other supportive equipment. Most elderly people did not bring their prescriptions and often could not remember their medical history, names of medications, or dosages of their medicine. Medical records are essential to understand the history of illnesses and treatments. For example, patients with diabetes require insulin or oral hypoglycemic drugs, asthma patients require beta-antagonists or corticosteroids, hypertension patients require antihypertensives, heart disease patients need beta-blockers or nitroglycerin preparations, and dialysis patients need potassium-exchanging resins, which is crucial for reducing potassium levels when dialysis access is limited. Over-the-counter medications such as fever or pain relievers, antihistamines, and sanitary products are also necessary. Supportive equipment is required, such as insulin injection supplies (vials, needles, or pens with replaceable insulin cartridges), glucometers, oxygen cylinders, and nebulizers. These should be considered part of the emergency kit to be taken along. Daily living aids should also be prepared, including wheelchairs, hearing aids, canes, walkers, dentures, glasses, and incontinence briefs. Disasters can disrupt access to healthcare facilities and pharmacies, making it challenging for the elderly to obtain essential medications and supplies. Disaster preparedness efforts should include measures to ensure continuous access to medication and medical supplies during emergencies. This may involve stockpiling medications, establishing mobile medical clinics, and providing home delivery services for elderly individuals who are unable to visit pharmacies. ^10, 11, 25^

### 2. Mobility assistance

Mobility assistance is one of the primary disaster preparedness needs of the elderly. Many elderly individuals experience reduced mobility owing to age-related physical limitations or chronic health conditions. During disasters such as hurricanes or wildfires, evacuation may be necessary to ensure safety. However, elderly individuals may face a challenging evacuation owing to mobility issues. Providing assistance with transportation and ensuring accessible evacuation routes are essential to address this need. Communities can establish transportation assistance programs and designate accessible evacuation routes to support elderly individuals in safe evacuation during emergencies. ^11^

### 3. Communication support

Communication support is essential to address disaster preparedness among the elderly. Elderly individuals may have hearing or vision impairments, which make it difficult for them to receive emergency alerts and information. Clear and accessible communication channels are necessary to ensure that the elderly receive timely information and instructions during emergencies. Communities can implement text-based alert systems, use visual cues in emergency communication, and provide outreach programs to support elderly individuals with communication challenges. ^18^

### 4. Social support

Social support networks are essential for the preparation of older adults for disasters. Building strong social support networks within communities can help ensure that elderly individuals have access to assistance and resources before, during, and after a disaster. Programs that connect elderly individuals with volunteers who can provide assistance with evacuation, medical care, and other needs can help enhance resilience and reduce vulnerability during emergencies. ^18, 24, 25^

### 5. Shelter and other facilities

The establishment of specialized shelters and facilities is essential to address the specific requirements of the elderly during disasters. Designating shelters and facilities equipped to accommodate elderly individuals, such as providing accessible accommodations, medical support services, and trained staff, can help ensure their safety and well-being during emergencies. These specialized facilities can provide a supportive environment for elderly individuals, who may require additional assistance or medical care during disasters. ^11, 25^

### 6. Education and training programs

Education and training programs tailored to the needs of the elderly are essential to enhance disaster preparedness. Providing education on evacuation procedures, emergency preparedness kits, and strategies for managing chronic health conditions during emergencies can empower elderly individuals to take proactive steps in preparing for disasters. Community-based training programs can help ensure that elderly individuals have the knowledge and skills needed to protect themselves and their loved ones during emergencies. ^22^

Beside that there are several factors that affect disaster preparedness in elderly such as:

### 1. Education

Education has greatly influenced disaster preparedness among elderly people. Many elderly people have low literacy levels and some of them cannot write, so they do not understand how to get information about disasters and help. Thus, education level serves as a barrier to disaster preparedness. ^20^

### 2. Disability

Hearing ability is also related to disaster preparedness literacy. In the study conducted by Bell et al. (2021), ^10^ participants with hearing impairments followed flood news by utilizing alternative methods such as visual alerts, text notifications, or assistance from others to access weather forecasts and flood warning announcements. Older people with hearing impairments cannot listen to the information being disseminated. This can affect the preparedness. However, no research has been conducted on the intersection of hearing abilities and disaster preparedness.

### 3. Income

Income plays a significant role in enabling elderly people to prepare for disasters. If they have no or a low income, they cannot meet their needs and cannot survive. Low financial resources make them unsure whether they will be capable of covering food and shelter expenses for at least one day during an evacuation. Based on research conducted by Adepoju et al. (2023), ^12^ a survey on survival disaster preparedness action and planning disaster preparedness action indicates that low-income elderly individuals tend to neglect survival actions. Meanwhile, the findings found by Fletcher et al. (2022) ^11^ showed that after experiencing disasters, the elderly population experienced worry and stress. This is because of low financial resources. They are not sure if they will be able to pay for food and shelter for one day in the event of an evacuation. According to Bell et al. (2021), ^9^ the elderly acknowledged that their options were unevacuated because of a lack of alternative housing options, financial constraints preventing temporary accommodation, and discomfort associated with evacuation shelters.

### 4. Communication and information technology

Common challenges encountered in emergency response include barriers to communication technologies. This communication technology can also be useful as a disaster-warning system. The lack of a disaster warning system will have a major impact. A case reported by Yulianto et al. (2020) ^17^ during the Palu earthquake disaster showed that impacted individuals did not receive any advance warning about the tsunami or liquefaction. Although some people received tsunami alerts through social media, they came too late for an effective response. Thus, the Early Warning System (EWS) holds significance in initial preparedness, potentially safeguarding lives, infrastructure, and public facilities from disaster impact. This proves that a warning system in Indonesia is still lacking.

Another issue that serves as a barrier is the lack of disaster training for the elderly. Insufficient training hinders local communities from voluntarily engaging in disaster risk management. The role of local governments in reaching out to the elderly through training or education on disasters is essential; otherwise, residents may underestimate the threat of disasters. Furthermore, communicating disaster risks to vulnerable elderly populations is often ineffective due to barriers related to literacy and age-related impairments, such as hearing and vision difficulties. ^21^

According to Bell et al. (2021), ^10^ the elderly admitted that most information regarding communication about disaster preparedness and available response resources often lacks relevance and simplicity. Despite the widespread use of smartphone apps, social media, and other online communication by various institutions and organizations for rapid information dissemination, these channels remain inaccessible to many seniors, especially those lacking internet access or mobile devices. This unequal distribution of disaster response resources contributes to prolonged and difficult recovery of vulnerable elderly individuals.

### 5. Family

Families play a pivotal role in providing primary support for older adults, aiding in disaster preparation and recovery. The family is the support of the elderly because the family is the one that provides shelter and other resources when elderly facing a disaster. Family members may also invite home care providers to offer formal caregiving support when they cannot physically reach the healthcare service. Family members provide communication between patients and healthcare providers, maintain continuity of care, and encourage essential medication adherence. ^13^

### 6. Access to health service

Access to healthcare facilities depends on factors such as distance, mobility facilitators and barriers, transportation options, road conditions, travel expenses, and other associated direct and indirect expenses, all of which are designed as inhibitor factors for the utilization of healthcare services. Elderly individuals who are injured and suffer from chronic diseases require support and assistance to access healthcare services. Elderly people often face difficulties in reaching healthcare services owing to poor road conditions and injuries sustained during disasters. However, participants without significant injuries reported accessing post-earthquake health care services through mobile clinics, hospitals, and health posts. Many participants had access to hospitals and health posts for basic health checkups and treatment. Nonetheless, for those who sustain injuries and experience mobility loss due to an earthquake, access to intensive care facilities located within a feasible distance becomes essential. ^23^

To improve the preparedness of the elderly to disaster, some interventions are needed that include the following:

### 1. Enhance disaster preparedness through food bar

One of the primary challenges in disaster management involves fulfilling the food requirements of vulnerable groups to meet their nutritional needs. In areas affected by disasters, the elderly more often experience malnutrition due to low energy, carbohydrate, protein, and fat intake. Fatmah et al. (2021) ^14^ attempted to create a food bar and recipe. This food bar is an emergency sustenance item made from locally obtained raw materials, engineered to be readily accessible while possessing increased economic worth owing to its specialized composition. Consuming 50 g of the broccoli-soybean-mangrove food bar per day (amounting to 246 calories) satisfies 14–16% of the daily caloric needs for older males and females, respectively, based on a recommended intake of 1800 calories for males and 1550 calories for females is enough for elderly. Therefore, this food bar serves as a viable alternative to emergency food options during disasters. Some dissatisfied perceptions of elderly disaster victims are that they think that the food bar is a staple food and not the main food. Beside that, when the food bar was tested, the researcher has not done the impact of the food to immune level of elderly. In addition, there is a need to fortify food with iron and prebiotics to provide more benefits to the health of the elderly.

### 2. Enhance disaster preparedness through education

Several researchers have conducted studies to improve disaster preparedness. Matsuo et al. (2021) ^30^ investigated the significance of disaster preparedness in mitigating isolation among elderly people. Elderly people in Japan prefer to isolate themselves when a disaster occurs rather than asking neighbors or other people for help. Therefore, they created a handbook, the “Disaster Preparedness Handbook,” and conducted a survey where the results indicated that the elderly population had a higher level of isolation precaution for themselves compared to the level of isolation precaution for others. Thus, education on disaster preparedness is needed to prevent isolation among the elderly. However, the study is limited by the age group selection and the closeness of the neighbors and the type of community of the participants, which makes a significant difference in the individual characteristics of the intervention and comparison groups who participated in the study. ^30^

Another study by Fatmah (2022) ^15^ in Indonesia provides education regarding the meaning of disasters, types of disasters, the impact of disasters, family actions before, during, and after disasters, and disaster prevention. After education, family knowledge has increased. Some families have prepared equipment such as emergency bags containing important documents, logistical supplies (e.g., food), and medication. There are limitations to this study, in which a control group was not included for comparison with the intervention group, in addition to its small sample size. ^15^

According to Yarmohammadian et al. (2023), ^27^ it is necessary to educate the CBHO (community-based health organizations) through the development of a curriculum/syllabus for CBHO training in serving the elderly when facing disasters. The knowledge provided by the CBHO is in accordance with the syllabus, which includes the concept of aging and analysis of the impact of disasters on the elderly, providing for the needs of the elderly, physical and mental analysis, how to coordinate with special organizations to handle the elderly, and the threats faced by the elderly after a disaster.

### 3. Enhance disaster preparedness through assistive technology

De Luna & Pingol (2020) ^26^ developed new technologies for help during disaster and emergencies namely “Help Bro.” This rescue application can serve government agencies, such as the Red Cross. Users can use the “Help Bro” application to alert others about impending dangers and help them avoid hazardous areas. There are several pages available in the application, for example, a page for registration for the user to enter personal information; a help page that enables the application to identify the user’s location and generate a report when a user requests assistance; and a user report page that allows the users to send requests for help to the rescue team. The rescue team’s address is displayed along with options to specify the type of assistance needed.

Nakai et al. (2022) ^19^ developed tools (K-DiPS) to help vulnerable people provide medical information for use in disasters. K-DiPS comprises two components: K-DiPS Solo, a smartphone application, and K-DiPS online, a web application specifically designed for disaster management purposes by local government authorities. K-DiPS Solo is used by filling in the personal information of medically vulnerable people (MVP), and local governments can access this application via K-DiPS online. The K-DiPS Solo includes essential information such as personal details (name, gender, date of birth), emergency contact, medical history, need for long-term care, support (doctor, home visit, etc.), activities of daily living (ADL), and support needed (meal, excretion, dressing, transferring, mobility, bathing, etc.). Thus, when an MVP needs help, the local government can provide necessary health items (medicines and medical equipment).

Fathoni et al. (2020) ^16^ through his research invented an application named “application Bromo alert.” The application has several features: weblogs that offer information about Mount Bromo, including its history and past eruption events; shelters that provide details about shelters, including building names, locations, and proximity to disaster-prone areas; healthcare facilities that contain information about healthcare facilities available in the area; vlogs that provide informative content, especially targeted towards foreign tourists; and CCTV, which allows the public to view live updates of current conditions, particularly the latest situation at Mount Bromo. ^16^

While various technologies have been developed to support disaster mitigation efforts, none are specifically tailored to meet the needs of elderly. A key limitation is that many elderly individuals lack access to mobile devices and the internet. This highlights the need for further research aimed at developing accessible and appropriate technologies to improve disaster preparedness among the elderly.

## Conclusions

The study’s findings highlighted the importance of preparedness in supporting the elderly, including aspects such as medication management, mobility assistance, communication and social support, shelter as well as education and training. However, several factors can hinder disaster preparedness, including education, income, disability, communication and information technology, family, and access to health services. To improve disaster preparedness among the elderly, discoveries have been made in emergency food, educational programs, health technology, support, and warning systems. Based on available literature, this is the first literature review that examines disaster preparedness among the elderly. Hopefully, it can provide important information for policymakers and for any further research.

## Data Availability

No data are associated with this article. Figshare: Natural Disaster and Medication Preparedness Among Elderly (DOI https://doi.org/10.6084/m9.figshare.27292446.v4). ^31^ This project contains the following underlying data:
•
Figure 1 Search Keywords •
Figure 2 Flow Diagram of Review Process •
Table 1 Inclusion Criteria Based on Scientific Database Used •
Table 2 List of Reviewed Name Journal •
Table 3 Result Study Data are available under the terms of the Creative Commons Attribution 4.0 International license (CC-BY 4.0). Figshare: Prisma Checklist for ‘Natural disaster and medication preparedness among elderly’ (DOI https://doi.org/10.6084/m9.figshare.27252945.v7). ^32^ Data are available under the terms of the Creative Commons Attribution 4.0 International license (CC-BY 4.0).
