# Supplementary material for: Natural disaster and medication preparedness among elderly: a scoping review
Source: F1000Res. 2025 Jun 2;13:1510. Originally published 2024 Dec 9. [Version 2] doi: 10.12688/f1000research.157483.2 (PMC12296270; doi:10.12688/f1000research.157483.2)
Supplement: Peer review history

# Reviewer report: Reviewer response for version 2

**DOI:** 10.5256/f1000research.181530.r389318

**Published:** 2025-07-26

**Authors:** Elena Valkanova

**Affiliations:** 1 Epidemiology and Disaster Medicine, Medical University -Plovdiv, Plovdiv, Bulgaria

The issue examined in this study is highly relevant.This paper could make a meaningful contribution to the field of disaster preparedness.

Are the rationale for, and objectives of, the Systematic Review clearly stated?

Partly

Is the statistical analysis and its interpretation appropriate?

Not applicable

If this is a Living Systematic Review, is the ‘living’ method appropriate and is the search schedule clearly defined and justified? (‘Living Systematic Review’ or a variation of this term should be included in the title.)

Not applicable

Are sufficient details of the methods and analysis provided to allow replication by others?

Partly

Are the conclusions drawn adequately supported by the results presented in the review?

Yes

Reviewer Expertise:

Disaster Medicine

I confirm that I have read this submission and believe that I have an appropriate level of expertise to confirm that it is of an acceptable scientific standard.

# Reviewer report: Reviewer response for version 2

**DOI:** 10.5256/f1000research.181530.r389826

**Published:** 2025-06-20

**Authors:** Annekathryn Goodman

**Affiliations:** 1 Harvard Medical School, Boston, MA, USA

This study review existing literature on the preparedness of elderly populations. Interestingly, there is very little written about this even though  the elderly are significantly impacted. The authors make great suggestions for interventions at the personal and municipal level. I would suggest adding a table  with these suggestions foe quick access.

Are the rationale for, and objectives of, the Systematic Review clearly stated?

Yes

Is the statistical analysis and its interpretation appropriate?

Not applicable

If this is a Living Systematic Review, is the ‘living’ method appropriate and is the search schedule clearly defined and justified? (‘Living Systematic Review’ or a variation of this term should be included in the title.)

Not applicable

Are sufficient details of the methods and analysis provided to allow replication by others?

Yes

Are the conclusions drawn adequately supported by the results presented in the review?

Yes

Reviewer Expertise:

gynecology and disaster medicine

I confirm that I have read this submission and believe that I have an appropriate level of expertise to confirm that it is of an acceptable scientific standard.

# Reviewer report: Reviewer response for version 1

**DOI:** 10.5256/f1000research.172936.r355519

**Published:** 2025-01-20

**Authors:** Ryan Maves

**Affiliations:** 1 Wake Forest University School of Medicine, Winston–Salem, North Carolina, USA

Thank you for the opportunity to review this manuscript. In this paper, the authors conducted a scoping review of disaster preparedness as it relates to elderly people. I think there is some interesting information in here, and some careful re-editing and re-organization of the text could hopefully make it stronger.

 General comment:

 The overall quality of written English needs improvement, with many grammatical errors and overly-long descriptions in the text.

 As a specific example, the opening three sentences of the main text could be re-written as “The global population of elderly people, defined as those 60 years of age or greater, is large and increasing. There were an estimated 1 billion elderly people alive in 2024, a group that is projected to grow to 1.4 billion in 2030 and 2.1 billion by 2050.” An explanation of how becoming elderly is a function of aging is not needed.

 Specific comments:

 Abstract – the results and conclusions are too brief; while the necessary brevity of an abstract constrains out ability to discuss results in enormous detail, some mention of the actual results of the review should be included.

 Introduction – It is not necessary to mention what ministries of health are, although it is useful to mention that disaster preparedness may fail to meet the needs of older people.

 Results – 5 papers out of 20 is not “most” (in reference to the number of papers that were written in the US). It is not necessary to list the journals of origin in the main text; this can be included in Table 3 and in the references.

 Results – The results section does not include any actual results, just a list of papers that met their inclusion criteria. Much of this information is included in the discussion but would be properly considered part of the results. The basic organization of preparedness categories in the discussion (mobility assistance, social support, etc.) is a reasonable one, however.

Are the rationale for, and objectives of, the Systematic Review clearly stated?

Partly

Is the statistical analysis and its interpretation appropriate?

Not applicable

If this is a Living Systematic Review, is the ‘living’ method appropriate and is the search schedule clearly defined and justified? (‘Living Systematic Review’ or a variation of this term should be included in the title.)

Not applicable

Are sufficient details of the methods and analysis provided to allow replication by others?

Partly

Are the conclusions drawn adequately supported by the results presented in the review?

Partly

Reviewer Expertise:

Disaster medicine, sepsis, emerging infectious diseases

I confirm that I have read this submission and believe that I have an appropriate level of expertise to state that I do not consider it to be of an acceptable scientific standard, for reasons outlined above.

# Reviewer report: Reviewer response for version 1

**DOI:** 10.5256/f1000research.172936.r351500

**Published:** 2025-01-06

**Authors:** Elena Valkanova

**Affiliations:** 1 Epidemiology and Disaster Medicine, Medical University -Plovdiv, Plovdiv, Bulgaria

The article “Natural disaster and medication preparedness among elderly: a scoping review” aims to explore disaster and medication preparedness among the elderly, including the prerequisites and the impediments to disaster preparedness as well as interventions to support their preparedness. This is highly relevant topic considering the growing disaster risk associated with climate change and the vulnerability of the studied population. The authors performed a literature review of original research articles on the topic. 20 eligible articles were identified and further analyzed. The main conclusion states that more efforts are needed to improve disaster preparedness of the older people. The elements needed for disaster preparedness are identified:

- Medication preparedness
- Mobility assistance
- Communication support
- Social support
- Shelter and other facilities
- Education and training programs

Factors affecting disaster preparedness are formulated:

- Education
- Disability
- Income
- Communication and information technology
- Family
- Access to health services

Interventions to improve preparedness are proposed:

- Enhance disaster preparedness through food bar
- Enhance disaster preparedness through education
- Enhance disaster preparedness through assistive technology

In my opinion this study provides valuable viewpoint on disaster preparedness of the older people. The information that is provided could be useful for policymakers and should fuel further research.

 My comments on the article are as follows:

- There are two objectives provided for the study: “This review aimed to explore all reported research and publications related to natural disasters and medication preparedness among the elderly” and “This review aims to explore disaster and medication preparedness among the elderly, including what the elderly need to prepare in facing disaster, as well as the factors that inhibit them from fulfilling the preparedness and certain interventions to support them to be more prepared”. The title is designed according to the first one but the content better matches the second one. 
- More comprehensive explanation of the search strategy could be given. The keywords are listed in Table 1 of the Supplementary Materials but the exact strategy is not quite clear and reproducible. Also, there are synonyms and related terms that are not captured by the keywords.
- Figure 2 from the Supplementary Materials could provide all numbers.
- Database and search approach states that the review was conducted following (PRISMA-ScR) guidelines. This guideline is meant to direct only the reporting not the conducting of a review.
- In the chapter “Enhance disaster preparedness through assistive technology” there is no information that two of the presented tools are assessed for being useful for older people. The third one reports that additional assistance could be needed by the older people for proper use of the application. As it is stated in the current research: “Despite the widespread use of smartphone apps, social media, and other online communication by various institutions and organizations for rapid information dissemination, these channels remain inaccessible to many seniors, especially those lacking internet access or mobile devices.” Could be noted that more specific research is needed in this otherwise very promising field.  

Minor comments:

- Some of the citations in the text have no corresponding number according to the reference list.
- In the paragraph: “Fathoni et al. (2020) through his research invented an application named “application Bromo alert.” The application has several features: weblogs that offer information about Mount Bromo, including its history and past eruption events; shelters that provide details about shelters, including building names, locations, and proximity to disaster-prone areas; healthcare facilities that contain information about healthcare facilities available in the area; vlogs that provide informative content, especially targeted towards foreign tourists; and CCTV, which allows the public to view live updates of current conditions, particularly the latest situation at Mount Bromo. ^20^” the reference that is cited is not number 20 but 16.
- Consider rephrasing the sentences to avoid reiteration: “To the best of our knowledge, this literature review is the first to examine disaster preparedness among the elderly preparedness towards disaster.”, “Two reviewers collaborated to develop an approach to search for and determine a suitable determination for the search.”, “The findings of this study need to be prepared to support disaster preparedness for the elderly (medication, mobility assistance, communication support, social support, shelter, education, and training).”.

In conclusion, the issue examined in this study is highly relevant and remains far from being resolved. I praise the authors for their efforts to enrich the scientific literature on the subject.

Are the rationale for, and objectives of, the Systematic Review clearly stated?

Partly

Is the statistical analysis and its interpretation appropriate?

Not applicable

If this is a Living Systematic Review, is the ‘living’ method appropriate and is the search schedule clearly defined and justified? (‘Living Systematic Review’ or a variation of this term should be included in the title.)

Not applicable

Are sufficient details of the methods and analysis provided to allow replication by others?

Partly

Are the conclusions drawn adequately supported by the results presented in the review?

Yes

Reviewer Expertise:

Disaster Medicine

I confirm that I have read this submission and believe that I have an appropriate level of expertise to confirm that it is of an acceptable scientific standard, however I have significant reservations, as outlined above.
